# Secondary Hemophagocytic Lymphocytosis in Inflammatory Bowel Disease

**DOI:** 10.3390/hematolrep17040033

**Published:** 2025-06-30

**Authors:** Jacob Boccucci, Ramalakshmi Thulluri, Chandini Kannan, Matthew Gold, Vamsi Kota

**Affiliations:** 1 Department of Medicine, Medical College of Georgia, Augusta University, Augusta, GA 30912, USA; ckannan@augusta.edu (C.K.); mgold@augusta.edu (M.G.); 2 Medical College of Georgia, Augusta University, Augusta, GA 30912, USA; rthulluri@augusta.edu; 3 Department of Hematology/Oncology, Medical College of Georgia, Augusta University, Augusta, GA 30912, USA; vkota@augusta.edu

**Keywords:** hemophagocytic lymphocytosis, pancytopenia, inflammatory bowel disease

## Abstract

Background and Clinical Significance: Hemophagocytic lymphohistiocytosis (HLH) is a rare and life-threatening condition that can go underdiagnosed due to overlapping features with severe infections. While the use of thiopurine in inflammatory bowel disease (IBD) has been associated with HLH, the majority of these patients will have a concurrent Epstein–Barr virus (EBV) infection. Case Presentation: This report presents a case of HLH in a patient previously treated with aza-thioprine for IBD without concurrent viral infection.

## 1. Introduction

Hemophagocytic lymphohistiocytosis (HLH) is a rare and life-threatening systemic hyperinflammatory syndrome characterized by an excessive activation of immune cells, namely cytotoxic T lymphocytes, macrophages, and natural killer (NK) cells. HLH is broadly classified into primary (familial) HLH and secondary forms. Primary HLH is associated with genetic mutations that affect cytotoxic lymphocytes with various defects in the perforin/granzyme cell-death pathway and typically presents in childhood [1]. In contrast, secondary HLH occurs due to an exaggerated immune response to various triggers such as infections, malignancies, or autoimmune diseases, and can affect individuals regardless of age [1,2,3]. In adult populations, it is often underdiagnosed, and the epidemiologic profile is not well defined. Though the average age of presentation is approximately 50, the incidence is not precisely known. One retrospective study over a 16-year period estimated that the syndrome accounted for as many as 1 in every 2000 inpatient admissions [4,5].

It is challenging to diagnose secondary HLH due to the variety of clinical and laboratory findings that often overlap with severe infections. According to the HLH-2004 diagnostic criteria, the diagnosis of HLH can be established if there is either a gene mutation found or if the patient presents with at least five of the eight clinical and laboratory findings: fever, splenomegaly, cytopenia, hypertriglyceridemia, hyperferritinemia, hemophagocytosis, diminished NK cell activity, or a high soluble CD25 (soluble interleukin-2 receptor) [6]. The HScore, another system developed to estimate the probability of HLH, is comprised of four clinical (known underlying immunosuppression, temperature, hepatomegaly, and splenomegaly), seven hematologic (hemoglobin, leukocyte, platelets, ferritin, triglyceride, fibrinogen, and aspartate aminotransferase (AST)), and one cytologic (hemophagocytic features on bone marrow aspirate) determinants, where a score ≥ 250 confers a 99% probability of HLH [7]. The syndrome is usually treated with immunosuppressants, etoposide, and allogeneic hematopoietic stem cell transplantation along with treating underlying triggers.

It has been described in the literature that there are several cases of association between HLH and inflammatory bowel disease (IBD). One study showed that in pediatric populations, there was more than a 100-fold increased risk of HLH in patients with IBD, and within that patient group, 80% of patients had a cytomegalovirus (CMV) at presentation [8]. The two main causes of HLH in this patient population are infections with CMV or Epstein–Barr virus, in which most patients are using immunosuppressive regimens that include thiopurines [9]. The patient reported in the following case, however, did not have a precipitating viral infection as the main triggering factor for HLH. The aim of this paper is to emphasize the importance of including HLH in the differential for a critically ill patient with a history of recently diagnosed IBD and toxic megacolon.

## 2. Case History

A 49-year-old female with known history of ulcerative colitis was initially transferred from an outside hospital due to concern for sepsis in the setting of toxic megacolon and transverse colon perforation. Prior to presentation for toxic megacolon, the patient had recently been treated for ulcerative colitis with azathioprine and infliximab; however, she did not experience any improvement in her condition. At the outside hospital, the patient had exploratory laparotomy along with subtotal colectomy ileostomy creation for toxic megacolon. Her hospital course was complicated by an intra-abdominal infection requiring multiple abdominal washouts and antibiotics. The patient’s hospital course was additionally complicated by enterocutaneous fistula in which multiple unsuccessful attempts were made to close the fistula. She ultimately required transfer to a tertiary medical center.

Upon arrival to the tertiary medical center, the patient was noted to be hypotensive, which improved with fluids. Due to concerns around sepsis, the patient was started on broad-spectrum antibiotics, including vancomycin, meropenem, and micafungin for a documented history of multidrug-resistant Pseudomonas. On initial physical exam, there was mucositis of the nares and lips, at which time dermatology was consulted for possible Stevens–Johnson syndrome. Dermatology evaluation determined that the patient’s condition was consistent with mucositis and related to her ulcerative colitis. Other considerations of the mucosal findings included reactive infectious mucocutaneous rupture secondary to mycoplasma, or herpes simplex virus infection (HSV); however, laboratory results of mycoplasma and HSV were negative.

The patient’s clinical course deteriorated with a sudden progressive hypotension with tachycardia requiring escalating doses of vasopressors. General surgery and gastroenterology were consulted in the setting of ulcerative colitis and enterocutaneous fistula with recommendation for continuing broad-spectrum antibiotics and supportive care. The patient’s clinical condition continued to worsen including the development of hypoglycemia requiring dextrose infusion and supraventricular tachycardia requiring adenosine. This was coupled with elevating lactic acidosis and a sudden episode of unresponsiveness which required intubation for airway protection and activation of Code Stroke team. Following intubation, the patient was started on Keppra for possible seizures, and continuous renal replacement for lactic acidosis. Additional work-up included a transthoracic echocardiogram consistent with Takotsubo cardiomyopathy requiring cariology consultation. Throughout the patient’s intensive care stay, she received treatment for septic and cardiogenic shock.

Based on the patient’s history of immunodeficiency in the setting of ulcerative colitis with azathioprine and infliximab therapy and concurrent infection, hemophagocytic lymphohistiocytosis was added to the differential. This differential was strengthened by daily laboratory data noting worsening cytopenia, repeated fevers, and evidence of organomegaly on CT imaging. Empirical dexamethasone therapy was considered for HLH. However, dexamethasone was held due to ongoing sepsis and concern for delayed wound healing of open abdominal wound. Hematology/oncology was consulted regarding concern for HLH and further treatment recommendation.

## 3. Investigation & Treatment

Initial hematology/oncology evaluation was concerning for hemophagocytic lymphohistiocytosis based on patient’s HScore of 273 (Table 1) along with markedly elevated interleukin 2 receptor of 5549. While initial recommendations were to obtain bone marrow biopsy early in the hospital course, this was not feasible based on the patient’s clinical condition. Additionally, given multiple ongoing infections and septic shock, empirical dexamethasone was deferred until disease confirmation by bone marrow biopsy. The ability to obtain bone marrow aspirate was complicated by the development of disseminated intravascular coagulopathy, driven by the patient’s sepsis. A hematology smear was without any dysplastic or malignant features upon review by pathologist. Additional laboratory data showed a negative viral panel, including cytomegalovirus and Epstein–Barr virus, but did have positive microbiology culture, including abdomen aspirate positive for pseudomonas contributing to septic shock.

Since development of dual-pressor shock on hospital day two, the patient had been receiving stress-dose steroids with hydrocortisone and fludrocortisone based on the guidelines for treatment of septic shock by the Society of Critical Care Medicine. Empirical dexamethasone had not been initiated due to the patient’s many comorbid conditions. On hospital day five, a joint decision amongst the patient’s family, critical care medicine team, and the bone marrow transplant team was made to transition steroid regimen to dexamethasone 10 mg/m^2^. Additionally, the initiation of etoposide to decrease the risk of death from progression of HLH was discussed with the patient’s primary decision maker. The primary decision maker was not agreeable to starting etoposide without a definitive diagnosis of HLH via bone marrow biopsy.

**Figure 1:**
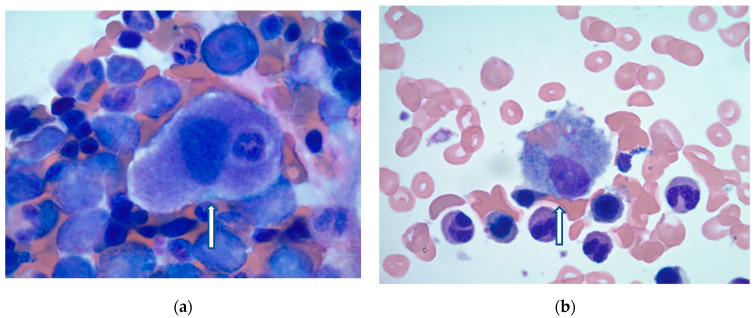
(**a**) Slide showing histiocytes displaying round contour with cytoplasmic projections which, combined with the other HLH-2004 criteria, give a diagnosis of HLH; (**b**) Activated histiocytes with expended cytoplasm and engulfed RBCs, lymphocytes, and PMN.

On hospital day 10, the patient’s condition improved, and bone marrow biopsy was obtained. Findings were consistent with HLH showing normocellular marrow with increased histiocytes and maturing trilineage hematopoiesis (Figure 1a,b). Given the patient’s clinical presentation, as well as laboratory and bone marrow findings, a diagnosis of secondary or reactive HLH was made by the pathologist. After confirmation of HLH, etoposide was again discussed with the patient’s primary decision maker. However, after patient-centered care discussions with the family, chemotherapy was withheld due to clinical improvement. Instead, the patient continued to be treated with a dexamethasone taper with significant clinical improvement. Overall, the patient had a 38-day hospital course with multiple complications, required tracheostomy, and was ultimately discharged to a long-term acute care hospital for continued recovery.

## 4. Discussion

Hemophagocytic Lymphohistiocytosis (HLH) is a rare and life-threatening syndrome known for causing systemic hyperinflammation and tissue damage, often leading to fatal outcomes. Based on the clinical features, imaging studies, exclusion of commonly seen infectious and malignant sources, and confirmatory bone marrow biopsy in the patient described in this case report, we believe that this was a case of secondary HLH triggered by her recent treatment course of azathioprine and infliximab for ulcerative colitis. While secondary HLH may arise from any number of conditions, it has largely been associated with infection, particularly in immunocompromised patients, such as those with inflammatory bowel disease taking immunosuppressants containing thiopurines [10]. However, these cases often occur in the setting of active infection with Cytomegalovirus (CMV) or Epstein–Barr virus (EBV). It is important to clarify that the patient described in the case above did have a positive microbiology culture for Pseudomonas in the setting of an open enterocutaneous fistula. This bacterium was thought to be the predominant driving force in the patient’s ongoing septic shock. As noted above, viral etiology had been ruled out with a negative panel that included the aforementioned viruses.

In a recent nationwide analysis comparing the prevalence of HLH among patients with and without inflammatory bowel disease (IBD), CMV was found to be the most common infectious trigger (16.0%), with lymphoma as the most associated malignancy (18.1%) [11]. Notably, a subset of these cases lacked a clear infectious or malignant trigger, which suggests multifactorial immune dysregulation due to the underlying disease or possibly the immunosuppressive therapy itself [11]. On review of current literature of HLH in IBD patients, those without viral triggers were extremely rare. Most published data are limited to case reports or small case series, restricting the authors’ ability to draw robust conclusions about causality or risk factors [12,13,14,15,16]. In addition to most cases predominantly occurring in the context of concomitant viral infections, they are also often documented in pediatric populations, further making it difficult to characterize the clinical phenotype, pathogenesis, and outcomes in this patient’s subgroup. Furthermore, these cases often lack a thorough diagnostic work-up to definitively exclude occult infection of viral etiology or malignancy as contributors, unlike the patient described in this case who underwent a comprehensive workup to rule out commonly associated triggers.

Diagnostic criteria for HLH are not specific to IBD populations, and the clinical overlap with other critical illnesses and inflammatory states, such as sepsis, complicates the interpretation, leading to underdiagnosis and/or misclassification. The HScore (see Table 1) is particularly useful in critically ill patients, where the clinical presentation may overlap with other hyperinflammatory syndromes, particularly when the HLH-2004 criteria are not fully met or are difficult to apply in adults [17,18,19,20]. The patient in this case above was also uncommon in severity of the disease, in the setting of multiple comorbidities and concomitant hyperinflammatory processes. Prior to confirmation of HLH on the bone marrow biopsy, the patient only met four out of eight B criteria, according to the HLH-2004 diagnostic criteria (fever > 38.5 °C, splenomegaly, cytopenia affecting ≥2 of 3 lineages, and soluble IL-2 receptor ≥ 2400 U/mL). While prolonged treatment courses of thiopurines have been thought to be an important hematologic insult in the pathogenesis of HLH, this pathophysiological mechanism remains incompletely understood [21]. New literature, recently published in the New England Journal of Medicine, suggests a threshold model, in which a combination of endogenous (genetic predisposition and baseline inflammation from IBD) and exogenous (immunosuppression, infection, malignancy) factors are thought to contribute to HLH, even in the absence of a viral trigger [17].

Finally, due to the patient’s rapid clinical deterioration, initial recommendations to obtain a bone marrow biopsy or start empiric standard of care treatment were deferred, uniquely altering the overall duration and treatment choice. According to HLH-2004 treatment recommendations, a regimen of etoposide, dexamethasone, and cyclosporine is utilized as a bridge to control hyperinflammation and improve survival until hematopoietic stem cell transplantation (HSCT) [17]. Given this patient’s marked co-morbidities, poor performance status, and lack of meaningful recovery, this case would likely not have met eligibility criteria for HSCT [17]. The decision to hold etoposide despite confirmed HLH was made due to clinical improvement on steroid therapy alone and family preference. This is a significant discrepancy from standard of care. The North American Consortium for Histiocytosis (NACHO) emphasizes prompt initiation of etoposide and dexamethasone as per the HLH-94/HLH-2004 protocols [12]. Initial therapy with dexamethasone alone and close monitoring may be considered in select less severe cases, but etoposide is often not delayed in critically ill or rapidly deteriorating patients like the one described above [12].

This case is an important and unique reflection on the consideration of HLH in the differential of critically ill patients. There is limited research available on patients with IBD and HLH without preceding viral infections or malignant triggers. Under-recognition in clinical practice and reporting bias in literature results in an overall underrepresentation of cases without a clear viral trigger. Additionally, published cases often lack detailed therapeutic and outcome data, further limiting evidence-based recommendations. Further research concerning the link between IBD and HLH, pertinent endogenous and exogenous factors as well as consideration of genetic testing could guide more specific diagnostic criteria and means of screening in this patient population. It may not be necessary to always involve Hematology/Oncology in all cases of IBD previously treated with thiopurine drugs. However, this case report highlights how heavy clinical suspicion in addition to the HLH-2004 criteria and the HScore can be a strong predictor of HLH, prompting consultation for further workup and treatment. This approach in future practice could lead to earlier diagnosis and therapy, ultimately allowing the medical community to reach a consensus on the optimal management of HLH in IBD patients without apparent viral or malignant triggers.

## Figures and Tables

**Table 1:** HScore for Reactive Hemophagoctyic Syndrome.

Findings		Score
Known Immunosuppression (long term immunosuppressive therapy)	Yes	18
Temperature (°F)	99.4 °F	0
Organomegaly	Hepatomegaly	23
Number of Cytopenia (Defined as hemoglobin ≤ 9.2 g/dL (≤5.71 mmol/L) and/or WBC ≤ 5000/mm^3^ and/or platelets ≤ 110,000/mm^3^)	3 lineages	34
Ferritin, ng/mL	>7500	50
Triglycerides, mg/dL	466	64
Fibrinogen, mg/dL	211	30
AST, U/L	37	19
Hemophagocytosis features on bone marrow aspirate	Yes	35
Total		273

## Data Availability

No new data were created or analyzed in this study. Data sharing is not applicable to this article.

## Abbreviations

The following abbreviations are used in this manuscript:

EBV	Epstein–Barr Virus
HLH	Hemophagocytic Lymphohistiocytosis
NK	Natural Killer
IBD	Inflammatory Bowel Disease
